# CAZyChip: dynamic assessment of exploration of glycoside hydrolases in microbial ecosystems

**DOI:** 10.1186/s12864-016-2988-4

**Published:** 2016-08-23

**Authors:** Anne Abot, Gregory Arnal, Lucas Auer, Adèle Lazuka, Delphine Labourdette, Sophie Lamarre, Lidwine Trouilh, Elisabeth Laville, Vincent Lombard, Gabrielle Potocki-Veronese, Bernard Henrissat, Michael O’Donohue, Guillermina Hernandez-Raquet, Claire Dumon, Véronique Anton Leberre

**Affiliations:** 1 Université de Toulouse, INSA, UPS, INP; LISBP, 135 Avenue de Rangueil, F-31077 Toulouse, France; 2 INRA, UMR792 Ingénierie des Systèmes Biologiques et des Procédés, F-31400 Toulouse, France; 3 CNRS, UMR5504, F-31400 Toulouse, France; 4 Centre National de la Recherche Scientifique, UMR 7257, F-13288 Marseille, France; 5 Architecture et Fonction des Macromolécules Biologiques, Aix-Marseille University, F-13288 Marseille, France; 6 INRA, USC 1408 AFMB, F-13288 Marseille, France; 7 Department of Biological Sciences, King Abdulaziz University, Jeddah, Saudi Arabia; 8 Laboratoire d’Ingénierie des Systèmes Biologiques et des Procédés (LISBP), UMR INSA/CNRS 5504/INRA 792, INSA Batiment Bio 5, 135, avenue de Rangueil, F-31077 Toulouse cedex 4, France

**Keywords:** CAZymes detection, Glycoside hydrolase, Microarray, Microbial functional diversity, Plant cell wall degradation, Transcriptomic analysis

## Abstract

**Background:**

Microorganisms constitute a reservoir of enzymes involved in environmental carbon cycling and degradation of plant polysaccharides through their production of a vast variety of Glycoside Hydrolases (GH). The CAZyChip was developed to allow a rapid characterization at transcriptomic level of these GHs and to identify enzymes acting on hydrolysis of polysaccharides or glycans.

**Results:**

This DNA biochip contains the signature of 55,220 bacterial GHs available in the CAZy database. Probes were designed using two softwares, and microarrays were directly synthesized using the in situ ink-jet technology. CAZyChip specificity and reproducibility was validated by hybridization of known GHs RNA extracted from recombinant *E. coli* strains, which were previously identified by a functional metagenomic approach. The GHs arsenal was also studied in bioprocess conditions using rumen derived microbiota.

**Conclusions:**

The CAZyChip appears to be a user friendly tool for profiling the expression of a large variety of GHs. It can be used to study temporal variations of functional diversity, thereby facilitating the identification of new efficient candidates for enzymatic conversions from various ecosystems.

**Electronic supplementary material:**

The online version of this article (doi:10.1186/s12864-016-2988-4) contains supplementary material, which is available to authorized users.

## Background

The degradation of polysaccharides such as cellulose, chitin, starch and glycogen is an essential feature of carbon cycle in the biosphere, a process that requires the contribution of various microorganisms that together deploy an arsenal of carbohydrate-degrading enzymes. Plant cell walls (PCWs) are composed of a composite network of macromolecules, including polysaccharides and lignin. The major polysaccharide in most plant cell walls is cellulose, which is composed of β-1,4 linked glucose polymers that interconnect through strong hydrogen bonds, forming crystalline microfibrils that are very stable. Cellulose is further embedded in a 3 D matrix composed of hemicelluloses, pectin and lignin [14] resistant to degradation. Compared to cellulose, hemicelluloses are heteropolymers that are variable in both chemical composition and structure, with heteroxylans and mannans being the two major categories of hemicelluloses in PCWs [4]. The exact compositional and structural features of hemicelluloses are dependent on a number of determinants, including the botanical origin of the plant, and also the pedoclimatic conditions prevailing at the time of growth [13, 14, 62]. Therefore, microorganisms that are responsible for biomass degradation are faced with a formidable task, which they achieve through the deployment of complex arsenals of enzymes [62].

Among the key PCW-degrading enzymes that are produced by microorganisms, the glycoside hydrolases (GH) and the carbohydrate esterases (CE) belong to a wide class of enzymes that modify, synthesize or hydrolyze carbohydrates: Carbohydrate Active enZymes, or CAZymes (ref CAZy). The CAZymes are prominent and highly diverse and have been identified in all taxa, representing typically 1–5 % of the predicted coding sequences in their genomes [39]. These proteins are expressed by microorganisms inhabiting almost all ecological niches (e.g., soil, marine environment and digestive tracts), where they participate in carbon cycling. The strategies of carbohydrate-degradation are often different at both the level of the microbial community and of individual microorganisms [30].

GH and CE can be encoded by multigenic operon-like clusters [45], such as Sus system [15, 51], that have been designated as Polysaccharide Utilization Loci in Bacteroidetes species [41, 44]. Evidence so far reveals that the proteins produced by such clusters display functional interplay with CAZyme components, displaying synergy on complex substrates [1, 48, 53]. In some anaerobic biomass-degrading bacteria, CAZymes, such as cellulases and hemicellulases, are arranged on cellulosomes, which are extracellular, cell-bound multi-enzyme complexes. In cellulosomes, the enzyme components are brought into close physical proximity, thus optimizing their synergistic actions and enhancing their biomass-degrading ability [3, 20].

GH and CE, and particularly those that are active on PCWs, are sought after for a wide range of industrial applications, including biorefining. In this field, the enzymes that are of particular interest include those active on cellulose (e.g., endoglucanases, EC 3.2.1.4, exoglucanases, EC 3.2.1.91 and EC 3.2.1.176) and on heteroxylans (e.g., endoxylanases, EC 3.2.1.8, β-D-xylosidases, EC 3.2.1.37 and α-L-arabinofuranosidases, EC; 3.2.1.55). Cellulose and hemicellulose yield monomeric sugars readily fermentable to produce alcohols, organic acids, or alkenes. The exploration of glycoside hydrolase (GH) diversity, and to a lesser extent CE can provide efficient biocatalysts and new insight into the different enzyme mechanisms that are used by microorganisms in biomass degradation. GHs have been used in many industries such as in paper production, textiles, detergents, feed and food [4, 33] as well as to promote healthy human nutrition and prevent diseases [17]. In the last decade, cellulases and more recently hemicellulases have been considered for biorefining [23, 30]. The discovery of GHs has been considerably accelerated with the metagenomic and metatranscriptomic approaches, which allow the identification of new enzymes in an unprecedented manner.

GH exploration is largely facilitated by the existence of the CAZy database (CAZy; www.cazy.org). This database describes the families of enzymes that catalyze the breakdown, biosynthesis or modification of carbohydrates and glycoconjugates. In the CAZy database, GHs are classified into families based on amino acid sequence similarities and others conserved features [7, 25, 26, 39]. GH- are classified in 135 families and represent approximately 47 % of the entire database. (April 2016) [7]. The vast majority of currently known GH are from bacterial origin.

DNA microarrays are widely used to profile gene expression and represent a relevant tool to study expression of key enzymes and monitor physiological changes of pure cultures or microbial communities [12, 18, 28, 42, 46, 50, 68]. This approach can also be useful to link microbial diversity to ecosystem processes and functions [22, 29, 67].

In this study, we developed the first microarray tool, termed CAZyChip, to quickly and accurately explore, at transcriptomic level, the GH composition of environmental samples. The CAZyChip provides snapshot views of the enzymes expressed by a single microorganism or more interestingly by microbial *consortia* derived from complex and various ecosystems. The biochip gives an opportunity to highlight enzyme cooperation along with the plant biomass degradation pathway. The present study demonstrates that the CazyChip represents a unique, robust and yet generic tool to dynamically analyze the expression of a large variety of GHs in parallel. The current version of this biochip allows the detection of 55,220 bacterial annotated GHs and contains the signatures of all bacterial GH in all families available to date in the CAZy database in addition to 53 CE sequences. The CAZy chip was validated using characterized enzymes from gut metagenomic libraries of different species, which were chosen for their known abilities to degrade plant cell walls. The encoding sequences of the enzymes of interest were recovered from microbiome of worm (*Pontoscolex corethrurus*), human, rumen, and termites these latter include fungus-growing (*Pseudacanthotermes militaris*), wood-feeding (*Nasutitermes corniger*), or soil-wood feeding (*Termes hispaniolae*). Furthermore, the developed biochip was tested to highlight the GH functional diversity of complex lignocellulolytic microbial communities, using a cow rumen-derived microbial consortium. The resulting biochip is able to test the GH functional diversity of complex microbial communities that present high metabolic and taxonomic diversity.

## Methods

### Custom microarray design

The design of oligonucleotides for the microarray was performed using either the Agilent e-Array online portal (https://earray.chem.agilent.com/earray/) or, when sequences were rejected by eArray, the ROSO software [16, 52]. When the design of 60-mers were impossible, a 40-mer or a pair of 25-mers associated with inert nucleotidic linkers was generated. For each targeted CAZyme gene (GH and CEs), three different 60-mer probes were designed and for each probe. The Agilent probe design algorithm assigned a BC score, which reflects uniqueness, secondary structure considerations, GC content and thermodynamic parameters, that predicts hybridization quality on the basis of their nucleotidic composition [19]. Five grades of BC scores were defined and indicated the quality of the designed probes. These different scores were, from the best to the worst: BC_1, BC_2, BC_3, BC_4 and BC_Poor. A total of 180,000 probes, including 4848 Agilent internal positive or negative control probes, were selected and synthesized in situ, on a glass slide using Agilent SurePrint technology to obtain a high-density DNA microarray tool on 4x180 K format (Agilent Technologies, Massy, France) [32]. The full description of the CAZyChip microarray has been deposited in the Gene Expression Omnibus (GEO) public database (GSE80173 study is at: http://www.ncbi.nlm.nih.gov/geo/query/acc.cgi?acc=GSE80173).

### Strains and growth conditions

Different GH cloned in plasmid or fosmid (pDest vector) were expressed by recombinant *E. coli* strains as previously described, [1, 2, 10, 34, 57, 59, 66]. Briefly, cultures were stopped at OD600nm between 0.4 and 0.6, and cells were harvested by centrifugation for 10 min at 5000 rpm at 4 °C. The supernatant was then discarded and the bacterial pellet immediately frozen at −80 °C before RNA extraction.

Microbial *consortia* analysis were performed on an anaerobic rumen-derived consortium RWS, which efficiently degrades lignocellulose, as reported by Lazuka et al. [36].

### Availability of materials section

The GH gene sequences used in this study were deposited under the GenBank accession number: TxAbf CAA76421; THSAbf ABZ10760; CfXyn AEA30147; TM1225 AAD36300.1; Abn43a and Pm08 CCO20984.1; Abn43b CCO20993.1; Abf51b CCO20994.1; Pm06 HF548274; Pm13 CCO21046.1, Pm14 CCO21057.1, Pm15 CCO21059.1; Pm21 CCO21105.1; Pm25 CCO21110.1; Pm31 CCO21136.1; Pm41 CCO21355.1; Pm43 CCO21392.1;Pm55 CCO21443.1; Pm65 CCO21487.1; Pm66 CCO21489.1; Pm69 CCO21492.1; Pm80 CCO21560.1; Pm81 CCO21564.1; Pm83 CCO21640.1; Pm85 CCO21658.1; and Pm87 CCO21793.1.

### RNA extraction

Bacterial pellets were lysed with 1 mg/ml lysozyme (Sigma-Aldrich, Isle d’Abeau Chesnes, France) for 5 min at 25 °C, followed by Total RNA extraction using the RNeasy Mini Kit (Qiagen, Courtaboeuf, France) according to the manufacturer’s recommendations. RNA concentration and purity was evaluated by measuring the absorbance ratio at 260/280 nm and 260/230 nm using a Nanodrop spectrophotometer (Labtech, Palaiseau, France). The Ratio Integrity Number (RIN) was evaluated using 2100 Bioanalyzer® (Agilent Technologies, Massy, France) and only samples with a RIN greater than 8 were hybridized on the microarray.

Total RNA of rumen derived *consortium* was extracted in two steps from nitrogen frozen samples using the PowerMicrobiome RNA isolation kit (MoBio Laboratories, Carlsbad, CA, USA) [36]. RNA purification was performed using AllPrep DNA/RNA minikit (Qiagen), according to the manufacturer’s recommendations.

### Labelling and amplification of total mRNA

The One-Color Low Input Quick Amp WT Labeling Kit™ (Agilent Technologies, Massy, France) was used to amplify and label 100 ng of RNA according to the manufacturer’s recommendations. The labelling efficiency was checked using a NanoDrop spectrophotometer operating at 260 nm to quantify cRNA and at 550 or 660 nm to measure cyanine 3 (Cy3) and cyanine 5 (Cy5) dye incorporation, respectively. Labeling efficiency was calculated as indicated by the manufacturer’s protocol (ratio cyanine quantity / amount of RNA) and was above 6.

### Microarray hybridization, washing and scanning

For each sample, 1650 ng of labeled and amplified cRNA was used for hybridization. The hybridization master mix was prepared according to manufacturer’s protocol (Agilent Technologies, Massy, France) and 100 μl were deposited onto a gasket slide, according to the Agilent Microarray Hybridization Chamber User Guide. Next, the active side of the microarray slide was placed on top of the gasket to form a properly aligned “sandwich slide pair”. The microarray slides were inserted into an Agilent Technology hybridization chamber then placed at 65 °C for 17 h with rotation at 10 rpm. After hybridization, the microarray was washed over a 1-min period, first using Gene Expression Wash Buffer 1 and then Gene Expression Wash Buffer 2 (Agilent Technologies, Massy, France) pre-warmed at 37 °C. After washing, the arrays were immediately scanned using an MS200 scanner (NimbleGen Roche Diagnostics, Meylan, France) with NimbleGen MS200 software v1.2 at 2 micron resolution.

### Data processing

The median signal of each spot in the hybridized arrays were determined and quantified using Feature Extraction software v11.5.1.1. The data from all the microarrays were normalized using the “limma” package function “normalizeQuantiles” and the “quantile” method [5, 56]. Normalization and statistical analyses of the data were performed using the Bioconductor packages (http://www.bioconductor.org) and R software v3.1.3. For each sample, the normalized fluorescence intensities of the three experimental replicates were analyzed and the mean values, standard deviations and correlation coefficients (%CV) were calculated. To determine whether probes were specific and target genes present, limma one way ANOVA test was carried out with False Discovery Rate adjusted *p value* < 0.05. Limma *t* test using “limma” package, was conducted to know in which comparison(s) this gene is differentially expressed (DE).

### Analysis of mRNA levels by qRT-PCR

One microgram of RNA was used as template to generate cDNA using the High Capacity cDNA reverse transcriptase kit (Applied Biosystems, Life Technologies, Saint Aubin, France). The reverse transcription reaction (20 μl final volume) was performed for 10 min at 25 °C, and then 2 h at 37 °C. Quantitative real-time PCR (qRT-PCR) assays were performed using SsoFast EvaGreen Supermix (Bio-Rad, Marnes-La-Coquette, France) on the StepOne instrument (Applied Biosystems, Life Technologies, Saint Aubin, France). Primers were validated by testing qRT-PCR efficiency using standard curves (95 % efficiency 105 %) as described previously [47]. Gene expression was quantified using the comparative Ct (threshold cycle) method. The RNA polymerase sigma S (*rpoS*) gene encoding the sigma factor sigma-38 was used as a reference to normalize the expression level of the targeted genes. Gene-specific primers sequences are described in Additional file 1: Table S1.

## Results

### Probe design

To design a generic microarray for the high-throughput detection of bacterial CAZymes mainly composed of GH’s, all of the bacterial GH protein sequences referenced in the CAZy database (www.cazy.org) up to January 2015 (133 families), were selected and their nucleotide sequences downloaded from the National Center for Biotechnology Information database (www.ncbi.nlm.nih.gov). We also selected sequences of interest obtained from human or termite guts and cow rumen metagenomic libraries created in our laboratory [2, 10]. The initial dataset used for probe design contained a total of 55,220 sequences and for each gene we designed three non-overlapping probes, with the aim to validate at least one probe per GH for use in a future prototype. With the e-array software, probe design has been possible on 55,012 sequences with a BC score attribution. This score reflects several criteria including the predicted hybridization quality, GC content and steric hindrances (Additional file 2: Table S2). A total of 56 % of probes displayed a BC_score of BC_1, 22 % of BC_2 reflecting the highest quality of predicted hybridization and a stable and consistent duplex with their targets. Only a small fraction of the probes were scored as BC_3 (11 %), BC_4 (11 %) and no BC_Poor were detected. Using the ROSO software we designed probes for the 208 of the remaining sequences.

The final CAZyChip was constructed using 180,000 probes, targeting 55,220 GHs able to detect 117 GH families on the 133 available in the CAZy database (www.cazy.org). We included 4848 positive and negative control probes. Non-bacterial families and GH7, 22 and 133, for which thermodynamical parameters did not provide specific probes, were not represented on the CAZyChip.

Regarding the high score of BC_1 and BC_2, we considered our CAZyChip as a promising high-density oligo-DNA microarray, which allows high throughput exploration of bacterial GHs.

### Validation of the CAZyChip

**Fig. 1:**
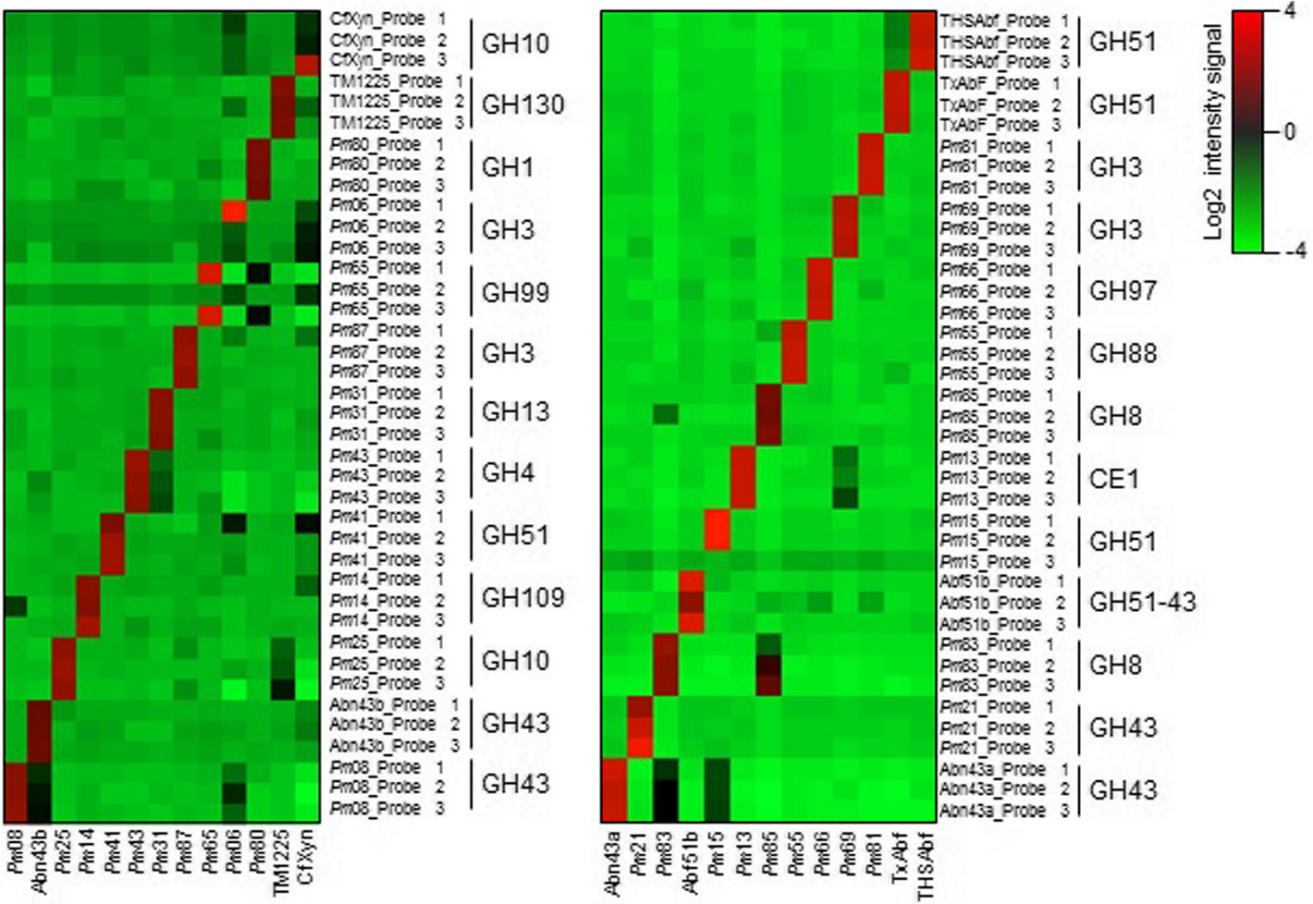
Heatmaps of log base 2 intensity signal of targeted probes for GHs cloned in plasmids samples. Each *horizontal line* represents a probe, and each vertical line represents an individual sample. Genes that were overexpressed are in *red*, whereas genes weakly expressed are in *green*. The color intensity indicates the degree of variation in expression

The specificity of the CAZychip probes was first evaluated using a set of plasmid bearing GH-encoding sequences, some of which encode well-characterized enzymes [1, 2, 10, 34, 57, 59]. To achieve this, 26 RNA samples from plasmid-bearing bacteria were labeled and hybridized with the probes on the CAZyChip. Figure 1 shows the heatmap (relative signal intensities) for this experiment and illustrates the fact that the vast majority of the samples hybridized quite specifically to the probes on the chip. *Pm*83 specific probes 2 and 3 not only hybridized with their target RNA, but also to a lesser extent with RNA from *Pm*85. This cross-hybridization can be easily explained by the fact that both *Pm*83 and 85 belong to GH8 family and share 81 % nucleotide sequence identity. Regarding probes specific for *Pm*65 (probe 2), *Pm*06 (probes 2 and 3), *Cf*Xyn (probes 1 and 2), and *Pm*15 (probe 3), these mostly failed to properly detect their target RNA in the test set (weak signals or no signal). Nevertheless, for each of these targets at least one probe proved to be adequate to properly hybridize to the target RNA and provide unambiguous detection.

**Fig. 2:**
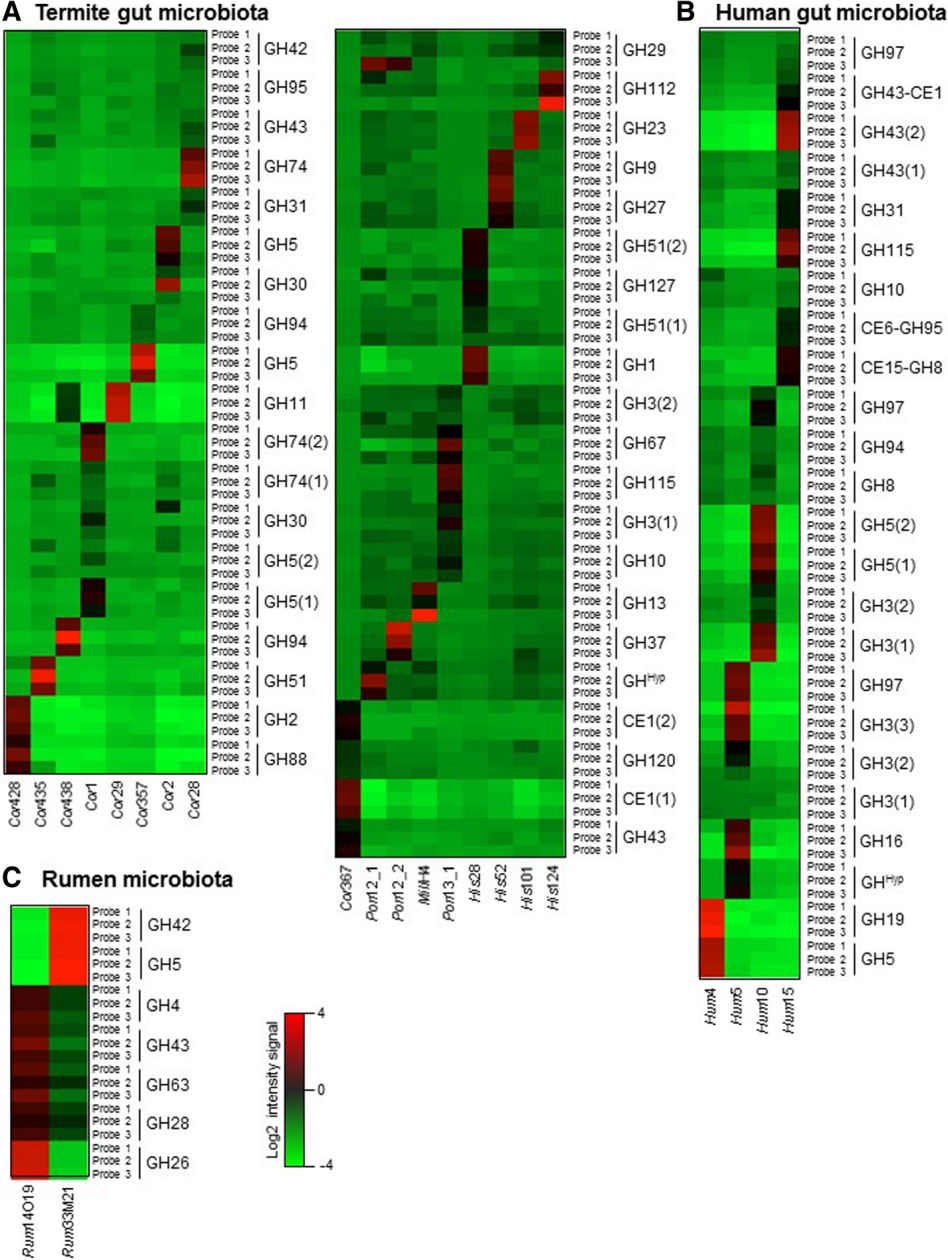
Heatmaps of log base 2 intensity signal of targeted probes for GHs cloned in fosmids **a** samples from termite microbiota labeled with cyanine 3 (*left panel*) or labeled with cyanine 5 (*right panel*), **b** samples from human microbiota and **c** samples from cattle rumen microbiota. Each horizontal line represents a probe, and each vertical line represents an individual sample. Genes that were overexpressed are in *red*, whereas genes weakly expressed are in *green*. The color intensity indicates the degree of variation in expression

To further validate the CAZyChip, RNA from 23 metagenomic clones derived from different gut microbial communities were used ([1, 2, 59, 61]; Table 1). These clones are all characterized by the fact that they bear more than one GH-encoding sequence, with at least one metagenomic clone containing up to 9 GH-encoding sequences (Additional file 3: Table S3). Upon hybridization with the CAZychip, the 23 metagenomic clones resulted in 69 positive signals (Fig. 2), which corresponds to a high detection rate. Most of the GH-encoding sequences were detected by at least one probe, but in some cases by two or three specific probes (Table 1). All genes were expressed in Rum33M21, or Cor367 whereas in Cor28 or Hum5 only a few genes were expressed (sequences GH3- and GH95- from the metagenomic clones Hum5 and Cor28 respectively were not detected), allowing identification of the gene responsible for the activity of each clone (Table 1 and Fig. 2a and b).

**Table 1:** List of metagenomic fosmid and their enzymatic activities highlighted by functional screening. GH’s listed have been included in the chip and the bold GH’s were detected on the CAZyChip

Name	Ecosystems	Structure of the carbohydrate compounds used for screening	GHs present on the CAZyCHip
*Cor*428	Termite gut *N. corniger*	Arabinofuranosidase	**GH88**, **GH2**
*Cor*435	Termite gut *N. corniger*	Arabinofuranosidase	**GH51**
*Cor*438	Termite gut *N. corniger*	Cellobiohydrolase	**GH94**
*Cor*1	Termite gut *N. corniger*	Arabinanase and Xylanase	**GH5**_(1)_, GH5_(2)_, **GH30**, GH74_(1)_, **GH74**_(2)_
*Cor*29	Termite gut *N. corniger*	Cellulase and Mannanase	**GH11**
*Cor*357	Termite gut *N. corniger*	Xyloglucanase and Cellulase	**GH5**, GH94
*Cor*2	Termite gut *N. corniger*	Arabinanase	**GH30**, **GH5**
*Cor*28	Termite gut *N. corniger*	Mannanase	**GH31**, **GH74**, **GH43**, GH95, **GH42**
*Cor*367	Termite gut *N. corniger*	Xylosidase	**GH43**, **CE1**_(1)_, GH120, **CE1**_(2)_
*Pon*12_1	Earth worm gut *P. corethruru*	Cellobiohydrolase	**GH** ^**Hyp**^
*Pon*12_2	Earth worm gut *P. corethruru*	β-glucanase	**GH37**
*Mili*H4	Termite gut *P. militaris*	Xylanase	**GH13**
*Pon*13_1	Earth worm gut *P. corethruru*	Xylosidase	**GH10**, **GH3**_(1)_, **GH115**, **GH67**, GH3_(2)_
*His*28	Termite gut *T.hispaniolae*	Arabinofuranosidase, Cellulase, Xyloglucanase, β-glucanase	**GH1**, GH51_(1)_, **GH127**, **GH51**_(2)_
*His*52	Termite gut *T.hispaniolae*	Cellulase	**GH27**, **GH9**
*His*101	Termite gut *T.hispaniolae*	Xylosidase	**GH23**
*His*124	Termite gut *T.hispaniolae*	Xylosidase, Cellulase	**GH112**, **GH29**
*Hum*4	Human feces	β-glucanase	**GH5**, **GH19**
*Hum*5	Human feces	β-glucanase	**GH** ^**Hyp**^, **GH16**, GH3_(1)_, GH3_(2)_, **GH3**_(3)_, **GH97**
*Hum*10	Human feces	β-glucanase	**GH3**_(1)_, **GH3**_(2)_, **GH5**_(1)_, **GH5**_(2)_, GH8, GH94, **GH97**
*Hum*15	Human feces	Xylanase	**CE15-GH8**, **CE6-GH95**, GH10, **GH115**, **GH31**, GH43_(1)_, **GH43**_(2)_, **GH43-CE1**, GH97
*Rum*14O19	Cow rumen	Mannanase	**GH26**, **GH28**, **GH63**, **GH43**, **GH4**
*Rum*33M21	Cow rumen	β-glucanase	**GH5**, **GH42**

Bold data correspond to GHs detected with the cazychip

Validation of the CAZyChip using individual GH-encoding sequences borne on multi-copy plasmids provided large amounts of RNA that procured strong, saturated hybridization signals for most of the specific probes. However, in the case of fosmid born sequences (metagenomics clones) the intensity of the different hybridization signals was variable, allowing us to determine an accurate minimal detection threshold. This threshold is defined as the minimum signal necessary to differentiate between positive and negative hits in a significant way. As in standard DNA Chip protocols, our samples were labeled with either Cy3 or Cy5. The minimal detection threshold was 8.00 (log 2 of intensity) for Cy3-labelled RNA and 6.70 (log base 2 of intensity) for Cy5-labeled samples. Calculation of the median of variation coefficients (CV) for all experimental probes revealed that this value lies in a narrow range from 1.43 and 4.75 % (Additional file 4: Figure S1), underlining the robustness of the CAZyChip. In addition, 14 GH-encoding sequences cloned either in plasmids (Uhbg_MP, TM1225, XylB, CfXyn and TxXyn) or in fosmids (*Cor428* and *Hum10*), were randomly chosen to be analyzed by qRT-PCR. The results of this analysis were consistent with those obtained using the CAZyChip (Additional file 5: Figure S2).

### Exploration of GH diversity evolution in microbial consortium from cow rumen

**Fig. 3:**
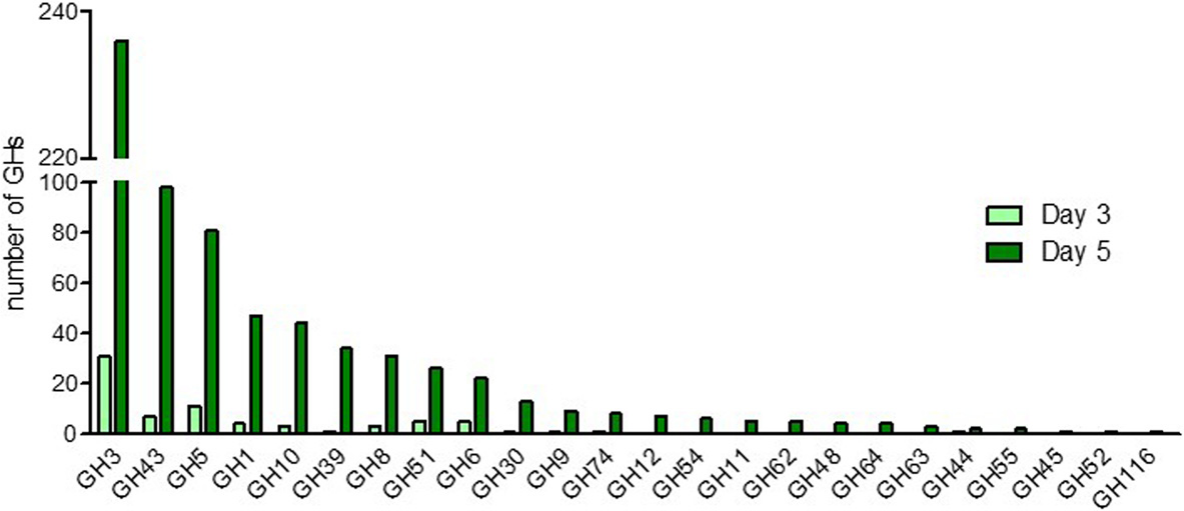
Expressed GH family known to be implicated in plant cell wall (PCW) degradation in day 3 and 5. For details see Additional file 6: Table S4

The CAZyChip was used to investigate the dynamic evolution of stable rumen-derived microbial community displaying good wheat straw degrading ability and a reduced complexity when compared to the parental inoculum [36]. Culture of this stable rumen-derived microbial community presented a 3-phase dynamic behavior over a 15 day period. The initial lag phase was characterized by stable, low-level enzyme activity and very little biomass degradation. The second phase (day 3 to 7), was characterized by an exponential burst of enzyme activities and the third phase was characterized by a stabilized level of enzyme activity [36]. The CAZyChip was used to compare two points that characterize the second phase of the culture, in order to highlight and identify what enzymes are the key players of the wheat straw degradation. The first point corresponded to the beginning of phase 2 (day 3), the second point was in the middle of the phase 2 (day 5), where enzymatic activities were high (Fig. 3).

A limma *t* test revealed that 2567 GHs were expressed in the two time points: day 3 and day 5 (Additional file 6: Table S4). Both samples displayed a common group of 257 expressed GHs. The two sample points also displayed GH expression unique to the specific time point, with the day 3 sample containing the expression of an additional GH belonging to the GH66 family (accession number AFH61494), and the day 5 sample containing expression of 2309 additional GH’s. Among the total 2566 GHs that were expressed at day 5, only 2 were down-regulated on day 5 compared to day 3 (Additional file 6: Table S4). The weighted differentially expressed genes, and those present at day 5, belong to 96 GH families and are displayed on Fig. 3.

Most of the differentially expressed genes encoding GHs are found in families that are correlated with either cellulose (e.g., GH1, GH3, GH5, and GH8) or hemicellulose (notably heteroxylan) hydrolysis (e.g., GH5, GH10, GH30, GH39, GH43, GH51) (in green Fig. 3b), is consistent with the known chemical composition of wheat straw [21, 35, 54]. CAZyChip analysis also revealed that GH arsenal deployed by the microorganisms in the rumen-derived microbial community contains an extensive range of GH families, including those related to starch hydrolysis (e.g., GH13) and others related to bacterial cell wall degradation (e.g., GH23; Fig. 3b), enzyme activities that are known to be highly represented in all kingdoms.

Using CAZyChip, we are able to explore expression of specific GH families implicated in the targeted functions of plant cell wall polysaccharide degradation. While focusing on GH families involved in enzymatic activities necessary to reach 25 % of wheat straw degradation [36], we observed an increase of the genes differentially expressed between day 3 and day 5, from GH families containing cellulase, xylanase, exoglucanase and beta-glucosidase activities in accordance with [36] (Table 2). We observed an enhanced expression of GH1, GH3 and GH5, which according to CAZy, some members of these families are beta-glucosidases and exoglucanases (for GH1 and GH3) or cellulases (for GH5) (Table 2). However, Lazuka et al. have previously shown enhanced cellulase and exoglucanase activities with a constant beta-glucanase activity [36]. Our results strongly suggest that enhanced GH5’s were implicated in efficient cellulase activity and that GH1 and GH3 explained the increased of exoglucanase activity. Our tool allows evaluation of the genetic potential of microbial consortium and highlights complementarity between GHs to contribute to these mechanisms of degradation of plant cell walls.

**Table 2:** Families of differentially expressed GH between in day 3 and 5 known to be implicated in plant cell wall (PCW) degradation and their enzymatic activities referenced in CAZy

Family	Number of DE genes	Enzymatic activities referenced in CAZy
GH3	236	ß-glucosidase; xylan 1,4-ß-xylosidase; ß-glucosylceramidase; ß-N-acetylhexosaminidase; a-L-arabinofuranosidase; glucan 1,3-ß-glucosidase; glucan 1,4-ß-glucosidase; isoprimeverose-producing oligoxyloglucan hydrolase; coniferin ß-glucosidase; exo-1,3-1,4-glucanase; ß-N-acetylglucosaminide phosphorylases
GH43	98	ß-xylosidase; a-L-arabinofuranosidase; arabinanase; xylanase; galactan 1,3-ß-galactosidase; a-1,2-L-arabinofuranosidase; exo-a-1,5-L-arabinofuranosidase; [inverting] exo-a-1,5-L-arabinanase; ß-1,3-xylosidase
GH5	81	endo-ß-1,4-glucanase / cellulase; endo-ß-1,4-xylanase; ß-glucosidase; ß-mannosidase; ß-glucosylceramidase; glucan ß-1,3-glucosidase; licheninase; exo-ß-1,4-glucanase / cellodextrinase; glucan endo-1,6-ß-glucosidase; mannan endo-ß-1,4-mannosidase; cellulose ß-1,4-cellobiosidase; steryl ß-glucosidase; endoglycoceramidase; chitosanase; ß-primeverosidase; xyloglucan-specific endo-ß-1,4-glucanase; endo-ß-1,6-galactanase; hesperidin 6-O-a-L-rhamnosyl-ß-glucosidase; ß-1,3-mannanase; arabinoxylan-specific endo-ß-1,4-xylanase; mannan transglycosylase
GH1	47	ß-glucosidase; ß-galactosidase; ß-mannosidase; ß-glucuronidase; ß-xylosidase; ß-D-fucosidase; phlorizin hydrolase; exo-ß-1,4-glucanase; 6-phospho-ß-galactosidase; 6-phospho-ß-glucosidase; strictosidine ß-glucosidase; amygdalin ß-glucosidase; prunasin ß-glucosidase; vicianin hydrolase; raucaffricine ß-glucosidase; thioglucosidase; ß-primeverosidase; isoflavonoid 7-O-ß-apiosyl-ß-glucosidase; ABA-specific ß-glucosidase; DIMBOA ß-glucosidase; ß-glycosidase; hydroxyisourate hydrolase
GH10	44	endo-1,4-ß-xylanase; endo-1,3-ß-xylanase; tomatinase; xylan endotransglycosylase
GH39	34	a-L-iduronidase; ß-xylosidase
GH8	31	chitosanase; cellulase; licheninase; endo-1,4-ß-xylanase; reducing-end-xylose releasing exo-oligoxylanase
GH51	26	endoglucanase; endo-ß-1,4-xylanase; ß-xylosidase; a-L-arabinofuranosidase
GH6	22	endoglucanase; cellobiohydrolase
GH30	13	endo-ß-1,4-xylanase; ß-glucosidase; ß-glucuronidase; ß-xylosidase; ß-fucosidase; glucosylceramidase; ß-1,6-glucanase; glucuronoarabinoxylan endo-ß-1,4-xylanase; endo-ß-1,6-galactanase; [reducing end] ß-xylosidase
GH9	9	endoglucanase; endo-ß-1,3(4)-glucanase / lichenase-laminarinase; ß-glucosidase; lichenase / endo-ß-1,3-1,4-glucanase; exo-ß-1,4-glucanase / cellodextrinase; cellobiohydrolase; xyloglucan-specific endo-ß-1,4-glucanase / endo-xyloglucanase; exo-ß-glucosaminidase
GH74	8	endoglucanase; oligoxyloglucan reducing end-specific cellobiohydrolase; xyloglucanase
GH12	7	endoglucanase; xyloglucan hydrolase; ß-1,3-1,4-glucanase; xyloglucan endotransglycosylase
GH54	6	a-L-arabinofuranosidase; ß-xylosidase
GH11	5	endo-ß-1,4-xylanase; endo-ß-1,3-xylanase
GH62	5	a-L-arabinofuranosidase
GH48	4	reducing end-acting cellobiohydrolase; endo-ß-1,4-glucanase; chitinase
GH64	4	ß-1,3-glucanase
GH63	3	processing a-glucosidase; a-1,3-glucosidase; a-glucosidase; mannosylglycerate a-mannosidase / mannosylglycerate hydrolase
GH44	2	endoglucanase; xyloglucanase
GH55	2	exo-ß-1,3-glucanase; endo-ß-1,3-glucanase
GH45	1	endoglucanase
GH52	1	β-xylosidase
GH116	1	ß-glucosidase; ß-xylosidase; acid ß-glucosidase/ß-glucosylceramidase; ß-N-acetylglucosaminidase

## Discussion

DNA microarray is one of the most popular technologies for gene expression profiling used in the past 15 years [28, 42, 46, 50, 68]. In this study we presented the development and the validation of the microarray CAZyChip dedicated to analyze the bacterial glycoside hydrolase expression. This is the first high throughput tool, based on DNA microarray technology, allowing the rapid characterization and exploration of the GHs arsenal of complex microbiota at the transcriptomic level. For design purposes, we first collected all sequences of bacterial GHs available in the CAZy data base, belonging to cultivated species, as well as some metagenomic sequences issued from uncultivated species. We then performed a probe bioinformatic design using eArray and ROSO softwares, which took into account the thermodynamics and specificity regardless of the secondary structures that probes can adopt. We validated probe specificity and the robustness of the biochip with different RNAs obtained from well characterized GHs cloned in plasmids and expressed in *E. coli*. For each GH, we validated at least one specific probe on the three designed per gene. For the great majority of GHs tested, the three probes gave a positive and specific hybridization signal, meaning that our probe design was highly effective.

Following this first validation step with unique GH overexpressed in bacteria, we studied the hybridization behavior of a series of metagenomic clones obtained from different metagenomic libraries. Metagenomic clones were selected for their enzymatic activity and can express up to 9 identified GHs. The CAZyChip allowed for the identification of genes responsible for the activity detected in each metagenomic clone. The multi-genic hybridization step allowed us to validate probes to identify 69 GHs. As an example, His28, which showed arabinofuranosidase activity, encodes two GH51 typical arabinofuranosidases, F but only one was expressed. 96 % of tested GHs had at least one validated probe. Previous studies have demonstrated that the use of multiple probes per target sequence is not essential for in situ synthesized 60mer oligonucleotides in bacterial Agilent’s arrays [37]. Our results demonstrate the robustness of the CAZyChip for GHs detection at transcriptomic level with experimental reproducibility.

Among naturally-occurring biomass-degrading systems, cow rumen represents a natural bioreactor. It is colonized by large communities of symbiotic microorganisms that produce an impressive arsenal of biomass-degrading enzymes, usually including cellulases and hemicellulases. With the CAZyChip, GH expression profiles at two different time points (day 3 and day 5) characterized by an exponential burst of enzyme activities were analyzed. At day 5, we identified overexpression of the GH families associated with cellulase (GH5, GH6, GH8, GH9 and GH48), xylanase (GH8, GH10), and exoglucanase (GH1, GH3) activities, which is in agreement with previous results [36]. The most common activities of GH3 include glucosidases, arabinofuranosidases, xylosidases and glucosaminidases and GH43 shows xylosidase, arabinofuranosidase, arabinanase, xylanase and galactosidase activities. Thus, these two families are implicated in degradation of arabinoxylan, the most abundant hemicellulose component in wheat straw [35] which explains the great number of genes overexpressed at day 5 in these GH families. An over representation of members of family GH13 and GH23 was seen, as they are implicated in common bacterial physiological processes and known to possess one of the broadest distributions among the gut microbiota [8, 17, 18]. It is the first time that such a generic tool is developed for GH detection from complex microbial ecosystems, although a custom microarray has been previously developed by El Kaoutari et al., to explore partial CAZome of specific human microbiota [17, 18]. This microarray contained probes targeting approximately 7000 genes encoding glycoside hydrolases and selected from 174 reference genomes from specific bacteria present in the human feces.

Our new CAZyChip tool allows the identification of an unprecedented amount of bacterial GHs (55,220) and few CEs, offering opportunities to study expression of a variety of GHs and combinations of enzymes in non-cultivable microorganisms found in any environment. The CAZychip provides an efficient method to explore complex environments, to analyze enriched niches for lignocellulose degradation, and to perform comparative studies. This transcriptomic screening approach (microarrays), reveals the genes that are being actively expressed by lignocellulolytic communities. This in turn allows us to consider the stability and/or performance of target enzymes, enabling the design of new enzyme cocktails and engineering microbial mixed cultures for an optimized lignocellulose bioconversion. Technologies for the rapid screening of GH’s activities are currently in development for high throughput analysis [6, 11, 38,  65]. Functional metagenomic has been proven to be useful tool to achieve this screening of GH’s activities (see review [27, 58]). However, like any screening related technology they face the paradigm “you get what you screened for”. In this context, the CAZyChip allows the observation of the enzymatic arsenal developed by microbial consortia on complex substrate, and could represent a decisive support before choosing a sample for further analysis.

As few CE sequences were included in the CAZyChip, in the near future, others CAZymes (i. e. glycosyltransferases, polysaccharide lyases, or auxiliary activities) could be detectable on the CAZyChip with the same approach. Thanks to its flexible design, this biochip will be able to accommodate additional probes [9] and could be upgradable, taking into account the regular updates of the CAZy database. Minty et al. have previously proven that fungal-bacterial consortia are efficient for the biosynthesis of valuable products from lignocellulosic feedstocks [43]. Probes for detection of this kind of CAZymes could easily be added on the CAZyChip, in order to highlight a large number of enzymes that work synergistically for cellulose and hemicelluloses breakdown [31, 40]. Understanding the biological process used by bacteria for carbohydrates depolymerization and metabolization is a considerable biotechnological interest not only for biorefineries but also to appreciate carbon flow in the environment, or to promote healthy human nutrition and prevent diseases [17, 18, 40]. The CAZyChip has been developed in a context of lignocellulosic biomass degradation but this biochip represents an excellent tool for other applications in the field of health and nutrition and more widely in any field interested in carbohydrate metabolism. Indeed, GHs are widely characterized in many biological systems such as human intestinal microbiota [17, 18] and the GHs profile are modified depending on eating habits and evolutionary plasticity of the human gut microbiome, playing a major role in nutrition and maintaining human health [24]. Modifications of their expression induce a number of diseases like colon cancer, Crohn’s disease, lactose malabsorption, food allergies, metabolic syndrome, type II diabetes, mucopolysaccharidoses [49, 55, 60, 64]. Thus applications referred for diagnostic or preventive health and nutrition could be explored, if considering GHs as biomarkers. Following glycosyltranferase expression could be also of great interest as they play an important role in the human antigenic system [63].

## Conclusion

In conclusion, the CAZychip developed in this study is a user-friendly, high-throughput, and reliable method to quickly explore GHs expression from complex environmental samples. It can be used to explore functional and ecological dynamics of the enzymatic machinery used by microbes for carbohydrate degradation. This approach can enhance the understanding of how the microbes metabolize polysaccharides and optimize polysaccharide or glycan deconstruction. The CAZyChip could guide the design of enzyme cocktails or the engineering of microbial mixed cultures for many applications.

## Abbreviations

CE, carbohydrate esterase; DE, differentially expressed; GH, glycoside hydrolase; PCR, polymerase chain reaction; PCW, plant cell wall; qRT-PCR, quantitative real-time PCR; RIN, Ratio Integrity Number

## Additional files

Additional file 1: Table S1. Primers sequences used for real-time qPCR quantification. (XLSX 12 kb) Additional file 2: Table S2. Summary of probes sequences on the CAZyChip, BC_scores and the accession number of associated gene. (XLSX 8992 kb) Additional file 3: Table S3. Nucleic sequences of fosmids from metagenomic study. (XLSX 57 kb) Additional file 4: Figure S1. Boxplots of coefficient of variation for specific probes of targeted GHs cloned in fosmids. (JPG 90 kb) Additional file 5: Figure S2. mRNA levels from (A) GHs cloned in plasmid (B-C) or in fosmids were quantified by real-time qPCR and normalized to rpoS mRNA levels. (JPG 43 kb) Additional file 6: Table S4. GHs differentially expressed in enriched microbial consortium from cattle rumen microbiome between day 3 and day 5. (XLSX 349 kb)

## References

[1] Arnal G, Bastien G, Monties N, Abot A, Anton Leberre V, Bozonnet S (2015). Investigating the function of an arabinan utilization locus isolated from a termite gut community. Appl Environ Microbiol.

[2] Bastien G, Arnal G, Bozonnet S, Laguerre S, Ferreira F, Fauré R (2013). Mining for hemicellulases in the fungus-growing termite Pseudacanthotermes militaris using functional metagenomics. Biotechnol Biofuels.

[3] Bayer EA, Belaich J-P, Shoham Y, Lamed R (2004). The cellulosomes: multienzyme machines for degradation of plant cell wall polysaccharides. Annu Rev Microbiol.

[4] Beg QK, Kapoor M, Mahajan L, Hoondal GS (2001). Microbial xylanases and their industrial applications: a review. Appl Microbiol Biotechnol.

[5] Bolstad BM, Irizarry RA, Astrand M, Speed TP (2003). A comparison of normalization methods for high density oligonucleotide array data based on variance and bias. Bioinformatics Oxf Engl.

[6] Boutard M, Cerisy T, Nogue P-Y, Alberti A, Weissenbach J, Salanoubat M (2014). Functional diversity of carbohydrate-active enzymes enabling a bacterium to ferment plant biomass. PLoS Genet.

[7] Cantarel BL, Coutinho PM, Rancurel C, Bernard T, Lombard V, Henrissat B (2009). The Carbohydrate-Active EnZymes database (CAZy): an expert resource for Glycogenomics. Nucleic Acids Res.

[8] Cantarel BL, Lombard V, Henrissat B (2012). Complex carbohydrate utilization by the healthy human microbiome. PLoS One.

[9] Carpita NC (2012). Progress in the biological synthesis of the plant cell wall: new ideas for improving biomass for bioenergy. Curr Opin Biotechnol.

[10] Cecchini DA, Laville E, Laguerre S, Robe P, Leclerc M, Doré J (2013). Functional metagenomics reveals novel pathways of prebiotic breakdown by human gut bacteria. PLoS One.

[11] Chauvigné-Hines LM, Anderson LN, Weaver HM, Brown JN, Koech PK, Nicora CD (2012). Suite of activity-based probes for cellulose-degrading enzymes. J Am Chem Soc.

[12] Chen X, Luo Y, Yu H, Sun Y, Wu H, Song S (2014). Transcriptional profiling of biomass degradation-related genes during Trichoderma reesei growth on different carbon sources. J Biotechnol.

[13] Chundawat SPS, Beckham GT, Himmel ME, Dale BE (2011). Deconstruction of lignocellulosic biomass to fuels and chemicals. Annu Rev Chem Biomol Eng.

[14] Cosgrove DJ (2005). Growth of the plant cell wall. Nat Rev Mol Cell Biol.

[15] D’Elia JN, Salyers AA (1996). Effect of regulatory protein levels on utilization of starch by Bacteroides thetaiotaomicron. J Bacteriol.

[16] Dugat-Bony E, Peyretaillade E, Parisot N, Biderre-Petit C, Jaziri F, Hill D (2012). Detecting unknown sequences with DNA microarrays: explorative probe design strategies. Environ Microbiol.

[17] El Kaoutari A, Armougom F, Gordon JI, Raoult D, Henrissat B (2013). The abundance and variety of carbohydrate-active enzymes in the human gut microbiota. Nat Rev Microbiol.

[18] El Kaoutari A, Armougom F, Leroy Q, Vialettes B, Million M, Raoult D (2013). Development and validation of a microarray for the investigation of the CAZymes encoded by the human gut microbiome. PLoS One.

[19] Ferraresso S, Vitulo N, Mininni AN, Romualdi C, Cardazzo B, Negrisolo E (2008). Development and validation of a gene expression oligo microarray for the gilthead sea bream (Sparus aurata). BMC Genomics.

[20] Fontes CMGA, Gilbert HJ (2010). Cellulosomes: highly efficient nanomachines designed to deconstruct plant cell wall complex carbohydrates. Annu Rev Biochem.

[21] Gilbert HJ (2010). The biochemistry and structural biology of plant cell wall deconstruction. Plant Physiol.

[22] Häkkinen M, Valkonen MJ, Westerholm-Parvinen A, Aro N, Arvas M, Vitikainen M (2014). Screening of candidate regulators for cellulase and hemicellulase production in Trichoderma reesei and identification of a factor essential for cellulase production. Biotechnol Biofuels.

[23] Hasunuma T, Okazaki F, Okai N, Hara KY, Ishii J, Kondo A (2013). A review of enzymes and microbes for lignocellulosic biorefinery and the possibility of their application to consolidated bioprocessing technology. Bioresour Technol.

[24] Hehemann J-H, Kelly AG, Pudlo NA, Martens EC, Boraston AB (2012). Bacteria of the human gut microbiome catabolize red seaweed glycans with carbohydrate-active enzyme updates from extrinsic microbes. Proc Natl Acad Sci U S A.

[25] Henrissat B (1991). A classification of glycosyl hydrolases based on amino acid sequence similarities. Biochem J.

[26] Henrissat B, Romeu A (1995). Families, superfamilies and subfamilies of glycosyl hydrolases. Biochem J.

[27] Heux S, Meynial-Salles I, O’Donohue MJ, Dumon C (2015). White biotechnology: state of the art strategies for the development of biocatalysts for biorefining. Biotechnol Adv.

[28] He Z, Deng Y, Van Nostrand JD, Tu Q, Xu M, Hemme CL (2010). GeoChip 3.0 as a high-throughput tool for analyzing microbial community composition, structure and functional activity. ISME J.

[29] He Z, Gentry TJ, Schadt CW, Wu L, Liebich J, Chong SC (2007). GeoChip: a comprehensive microarray for investigating biogeochemical, ecological and environmental processes. ISME J.

[30] Himmel ME, Bayer EA (2009). Lignocellulose conversion to biofuels: current challenges, global perspectives. Curr Opin Biotechnol.

[31] Himmel ME, Ding S-Y, Johnson DK, Adney WS, Nimlos MR, Brady JW (2007). Biomass recalcitrance: engineering plants and enzymes for biofuels production. Science.

[32] Hughes TR, Mao M, Jones AR, Burchard J, Marton MJ, Shannon KW (2001). Expression profiling using microarrays fabricated by an ink-jet oligonucleotide synthesizer. Nat Biotechnol.

[33] Kirk O, Borchert TV, Fuglsang CC (2002). Industrial enzyme applications. Curr Opin Biotechnol.

[34] Ladevèze S, Tarquis L, Cecchini DA, Bercovici J, André I, Topham CM (2013). Role of glycoside phosphorylases in mannose foraging by human gut bacteria. J Biol Chem.

[35] Lagaert S, Pollet A, Courtin CM, Volckaert G (2014). β-xylosidases and α-L-arabinofuranosidases: accessory enzymes for arabinoxylan degradation. Biotechnol Adv.

[36] Lazuka A, Auer L, Bozonnet S, Morgavi DP, O’Donohue M, Hernandez-Raquet G (2015). Efficient anaerobic transformation of raw wheat straw by a robust cow rumen-derived microbial consortium. Bioresour Technol.

[37] Leiske DL, Karimpour-Fard A, Hume PS, Fairbanks BD, Gill RT (2006). A comparison of alternative 60-mer probe designs in an in-situ synthesized oligonucleotide microarray. BMC Genomics.

[38] Liu G, Qin Y, Li Z, Qu Y (2013). Development of highly efficient, low-cost lignocellulolytic enzyme systems in the post-genomic era. Biotechnol Adv.

[39] Lombard V, Golaconda Ramulu H, Drula E, Coutinho PM, Henrissat B (2014). The carbohydrate-active enzymes database (CAZy) in 2013. Nucleic Acids Res.

[40] Lynd LR, Weimer PJ, van Zyl WH, Pretorius IS (2002). Microbial cellulose utilization: fundamentals and biotechnology. Microbiol Mol Biol Rev MMBR.

[41] Martens EC, Koropatkin NM, Smith TJ, Gordon JI (2009). Complex glycan catabolism by the human gut microbiota: the Bacteroidetes Sus-like paradigm. J Biol Chem.

[42] Maruyama K, Yamaguchi-Shinozaki K, Shinozaki K (2014). Gene expression profiling using DNA microarrays. Methods Mol Biol Clifton NJ.

[43] Minty JJ, Singer ME, Scholz SA, Bae C-H, Ahn J-H, Foster CE (2013). Design and characterization of synthetic fungal-bacterial consortia for direct production of isobutanol from cellulosic biomass. Proc Natl Acad Sci.

[44] Musso G, Gambino R, Cassader M (2011). Interactions between gut microbiota and host metabolism predisposing to obesity and diabetes. Annu Rev Med.

[45] Park BH, Karpinets TV, Syed MH, Leuze MR, Uberbacher EC (2010). CAZymes Analysis Toolkit (CAT): web service for searching and analyzing carbohydrate-active enzymes in a newly sequenced organism using CAZy database. Glycobiology.

[46] Patro JN, Ramachandran P, Lewis JL, Mammel MK, Barnaba T, Pfeiler EA (2015). Development and utility of the FDA ‘GutProbe’ DNA microarray for identification, genotyping and metagenomic analysis of commercially available probiotics. J Appl Microbiol.

[47] Pfaffl MW (2001). A new mathematical model for relative quantification in real-time RT-PCR. Nucleic Acids Res.

[48] Purushe J, Fouts DE, Morrison M, White BA, Mackie RI, North American Consortium for Rumen Bacteria (2010). Comparative genome analysis of Prevotella ruminicola and Prevotella bryantii: insights into their environmental niche. Microb Ecol.

[49] Qin J, Li Y, Cai Z, Li S, Zhu J, Zhang F (2012). A metagenome-wide association study of gut microbiota in type 2 diabetes. Nature.

[50] Rasooly A, Herold KE (2008). Food microbial pathogen detection and analysis using DNA microarray technologies. Foodborne Pathog Dis.

[51] Reeves AR, Wang GR, Salyers AA (1997). Characterization of four outer membrane proteins that play a role in utilization of starch by Bacteroides thetaiotaomicron. J Bacteriol.

[52] Reymond N, Charles H, Duret L, Calevro F, Beslon G, Fayard J-M (2004). ROSO: optimizing oligonucleotide probes for microarrays. Bioinformatics Oxf Engl.

[53] Rogowski A, Briggs JA, Mortimer JC, Tryfona T, Terrapon N, Lowe EC, Baslé A, Morland C, Day AM, Zheng H, Rogers TE, Thompson P, Hawkins AR, Yadav MP, Henrissat B, Martens EC, Dupree P, Gilbert HJ, Bolam DN (2015). Glycan complexity dictates microbial resource allocation in the large intestine. Nat Commun.

[54] Scheller HV, Ulvskov P (2010). Hemicelluloses. Annu Rev Plant Biol.

[55] Sheng YH, Hasnain SZ, Florin THJ, McGuckin MA (2012). Mucins in inflammatory bowel diseases and colorectal cancer. J Gastroenterol Hepatol.

[56] Smyth GK, Michaud J, Scott HS (2005). Use of within-array replicate spots for assessing differential expression in microarray experiments. Bioinformatics Oxf Engl.

[57] Song L, Siguier B, Dumon C, Bozonnet S, O’Donohue MJ (2012). Engineering better biomass-degrading ability into a GH11 xylanase using a directed evolution strategy. Biotechnol Biofuels.

[58] Steele HL, Jaeger K-E, Daniel R, Streit WR (2009). Advances in recovery of novel biocatalysts from metagenomes. J Mol Microbiol Biotechnol.

[59] Tasse L, Bercovici J, Pizzut-Serin S, Robe P, Tap J, Klopp C (2010). Functional metagenomics to mine the human gut microbiome for dietary fiber catabolic enzymes. Genome Res.

[60] Turnbaugh PJ, Hamady M, Yatsunenko T, Cantarel BL, Duncan A, Ley RE (2009). A core gut microbiome in obese and lean twins. Nature.

[61] Ufarté L, Laville É, Duquesne S, Potocki-Veronese G (2015). Metagenomics for the discovery of pollutant degrading enzymes. Biotechnol Adv.

[62] Van Dyk JS, Pletschke BI (2012). A review of lignocellulose bioconversion using enzymatic hydrolysis and synergistic cooperation between enzymes–factors affecting enzymes, conversion and synergy. Biotechnol Adv.

[63] Vasconcelos-Dos-Santos A, Oliveira IA, Lucena MC, Mantuano NR, Whelan SA, Dias WB (2015). Biosynthetic machinery involved in aberrant glycosylation: promising targets for developing of drugs against cancer. Front Oncol.

[64] Veneault-Fourrey C, Commun C, Kohler A, Morin E, Balestrini R, Plett J (2014). Genomic and transcriptomic analysis of Laccaria bicolor CAZome reveals insights into polysaccharides remodelling during symbiosis establishment. Fungal Genet Biol.

[65] Vidal-Melgosa S, Pedersen HL, Schückel J, Arnal G, Dumon C, Amby DB (2015). A new versatile microarray-based method for high throughput screening of carbohydrate-active enzymes. J Biol Chem.

[66] Vincentelli R, Cimino A, Geerlof A, Kubo A, Satou Y, Cambillau C (2011). High-throughput protein expression screening and purification in Escherichia coli. Methods San Diego Calif.

[67] Wu L, Thompson DK, Li G, Hurt RA, Tiedje JM, Zhou J (2001). Development and evaluation of functional gene arrays for detection of selected genes in the environment. Appl Environ Microbiol.

[68] Zhou A, He Z, Qin Y, Lu Z, Deng Y, Tu Q (2013). StressChip as a high-throughput tool for assessing microbial community responses to environmental stresses. Environ Sci Technol.

