# Supplementary figures and images for: CAZyChip: dynamic assessment of exploration of glycoside hydrolases in microbial ecosystems
Source: BMC Genomics. 2016 Aug 23;17(1):671. doi: 10.1186/s12864-016-2988-4 (PMC4994258; doi:10.1186/s12864-016-2988-4)

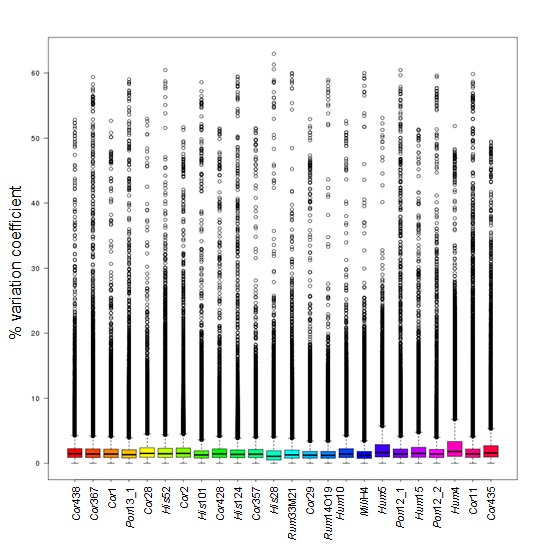

Supplement: Additional file 4: Figure S1. — Boxplots of coefficient of variation for specific probes of targeted GHs cloned in fosmids. (JPG 90 kb) [file 12864_2016_2988_MOESM4_ESM.jpg]

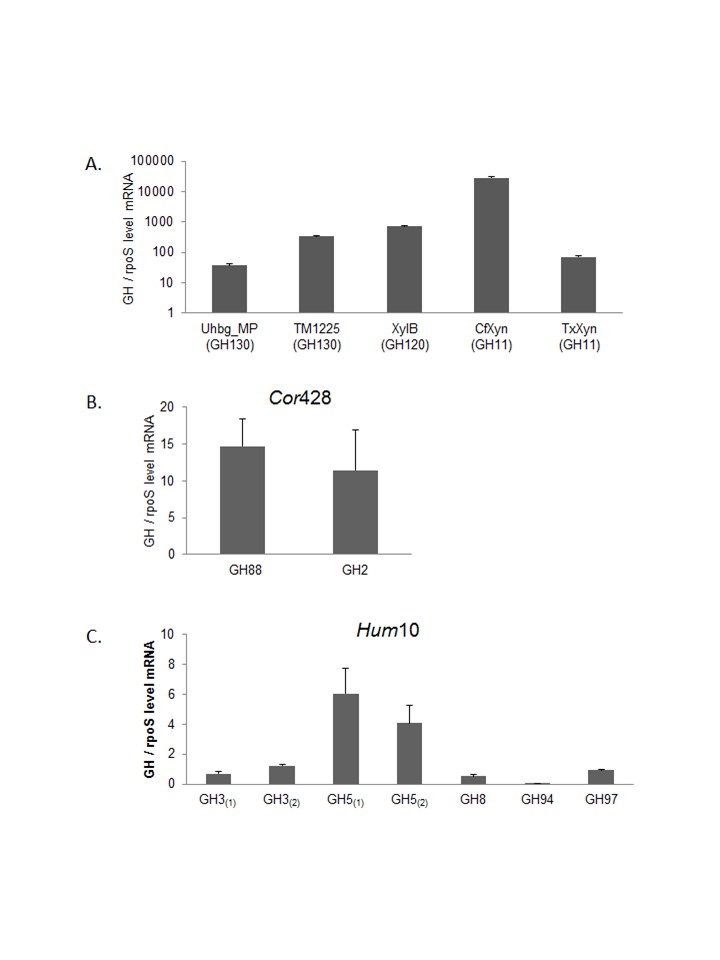

Supplement: Additional file 5: Figure S2. — mRNA levels from (A) GHs cloned in plasmid (B-C) or in fosmids were quantified by real-time qPCR and normalized to rpoS mRNA levels. (JPG 43 kb) [file 12864_2016_2988_MOESM5_ESM.jpg]
